# Discovery of a Potent Antimicrobial Peptide Through Rational Design: A New Frontier in Pathogen Control

**DOI:** 10.3390/biom15070989

**Published:** 2025-07-11

**Authors:** Bruna Agrillo, Monica Ambrosio, Rosa Luisa Ambrosio, Marta Gogliettino, Marco Balestrieri, Alessandra Porritiello, Maria Francesca Peruzy, Andrea Mancusi, Luigi Nicolais, Gianna Palmieri

**Affiliations:** 1 Institute of Biosciences and BioResources, National Research Council (IBBR-CNR), 80131 Naples, Italy; bruna.agrillo@ibbr.cnr.it (B.A.); monica.ambrosio@ibbr.cnr.it (M.A.); marta.gogliettino@cnr.it (M.G.); marco.balestrieri@cnr.it (M.B.); alessandra.porritiello@ibbr.cnr.it (A.P.); 2 Department of Veterinary Medicine and Animal Production, University of Naples Federico II, 80137 Naples, Italy; rosaluisa.ambrosio@unina.it (R.L.A.); mariafrancesca.peruzy@unina.it (M.F.P.); 3 Department of Food Microbiology, Istituto Zooprofilattico Sperimentale del Mezzogiorno, 80055 Portici, Italy; andrea.macusi@izsmportici.it; 4 Materias Srl, 80146 Naples, Italy; nicolais@unina.it

**Keywords:** cationic antimicrobial peptide, peptide design, antimicrobial activity, antibiofilm activity, ESKAPE pathogens, antimicrobial resistance

## Abstract

The increasing circulation of multi-drug-resistant pathogens, coupled with the sluggish development of new antibiotics, is weakening our capacity to combat human infections, resulting in elevated death tolls. To address this worldwide crisis, antimicrobial peptides (AMPs) are viewed as promising substitutes or adjuvants for combating bacterial infections caused by multidrug-resistant organisms. Here, the antimicrobial activity and structural characterization of a novel 13-amino acid cationic peptide named RKW (RKWILKWLRTWKK-NH2), designed based on known AMPs sequences and the identification of a key tryptophan-rich structural motif, were described. RKW displayed a broad-spectrum and potent antimicrobial and antibiofilm activity against Gram-positive and Gram-negative pathogens, including ESKAPE bacteria and fungi with minimal inhibitory concentrations (MBC) ranging from 5 µM to 20 μM. Structural results by fluorescence and Circular Dichroism (CD) spectroscopy revealed that the peptide was folded into a regular α-helical conformation in a membrane-like environment, remaining stable in a wide range of pH and temperature for at least 48 h of incubation. Furthermore, RKW showed low toxicity in vitro against mammalian fibroblast cells, indicating its potential as a promising candidate for the development of new antimicrobial or antiseptic strategies.

## 1. Introduction

The escalating threat of bacterial resistance to antibiotics and the consequent surge in infectious diseases caused by antimicrobial-resistant (AMR) bacteria have triggered a global health crisis, evoking a stark and alarming resemblance to the pre-antibiotic era [1,2]. Currently, the urgent development of novel antimicrobial agents is crucial to combat both susceptible microorganisms and antibiotic-resistant ‘superbugs’. Among these, the “ESKAPE” pathogens, encompassing *Enterococcus faecium*, *Staphylococcus aureus*, *Klebsiella pneumoniae*, *Acinetobacter baumannii*, *Pseudomonas aeruginosa*, and *Enterobacter* species, remain the leading cause of hospital-acquired infections, significantly increasing patient mortality. These bacteria successfully evade the effects of commonly used antibacterial drugs, resulting in a dramatic economic burden due to the prolonged hospital stays and reduced workforce productivity [3,4,5,6]. The World Health Organization has warned that if the antibiotic resistance persists at its current rate, it could lead to 10 million deaths yearly by 2050. Therefore, in the last decade, pharmaceutical companies have faced significant challenges in developing new antibiotic drugs, stemming from factors such as the high cost of research and the relatively low return on investment compared to other drug classes [7]. Reflecting its evolutionary antiquity, the innate immune system serves as the first line of defense, preceding the more refined acquired or adaptive immune response [8,9]. This system is critical for protecting the host from bacteria, viruses, fungi, parasites, and other infectious agents, by employing conserved recognition mechanisms. Pre-existing defense mechanisms enable this ancient system to provide rapid and non-specific protection against invading pathogens [8,9]. Among the diverse components of innate immunity, antimicrobial peptides (AMPs) are integral and potent effectors [10]. These peptides not only participate in the modulation of inflammatory responses, immune system functions, and apoptotic pathways, but also exhibit a broad spectrum of antimicrobial activities against various microorganisms [11,12,13,14]. Moreover, AMPs play a crucial role in inhibiting bacterial biofilm formation through various mechanisms, including disruption of the extracellular matrix (ECM) and interference with quorum sensing (QS). These mechanisms are particularly significant in combating multidrug-resistant pathogens, where biofilms contribute to persistent infections and increased antibiotic resistance [15,16].

At present, more than 3300 natural AMPs derived from different microorganisms, plants, mammals, amphibians, and insects have been registered in the Antimicrobial Peptide Database [17]. These molecules are short amphipathic peptides (7 to 50 amino acids), containing multiple hydrophobic and basic residues, and exhibiting a wide range of biological effects towards different pathogens. Moreover, AMPs are commonly known for their non-receptor-mediated membranolytic activity, which makes the development of bacterial resistance more challenging than those experimentally observed with conventional antibiotics [12,18,19]. The diversity of AMPs is extensive, leading to a broad categorization based on the secondary structure. A general classification includes α-helical peptides, β-sheet peptides, and hybrid peptides, which combine elements of both [20,21]. A common feature observed in many, though not all, antimicrobial peptides, across diverse structural families, is the clustering of cationic and hydrophobic amino acids into distinct domains, resulting in an amphipathic structure, which is crucial for their interaction with microbial membranes [22,23]. However, a significant subset of these molecules adopts an amphipathic α-helical structure only upon interaction with cell membranes. This specific behavior, coupled with their intrinsic physicochemical properties, enables AMPs to initially bind to anionic bacterial membranes through electrostatic interactions. Next, hydrophobic interactions facilitate their insertion into the lipid bilayer, leading to increased membrane permeability, the disassembly of the cell membrane, and ultimately, membrane disruption and cell lysis [24].

Therefore, AMPs have generated considerable interest as excellent and alternative therapeutic agents, and many efforts have been focused on developing novel AMPs with more potent activity [25,26]. To this aim, numerous studies have been performed by altering the original sequences of natural AMPs via different approaches, including amino acid substitution at one or more positions, truncation, deletion, or cyclization [27]. Such strategies lead to identifying those residues or structural determinants that are important in modulating the antimicrobial activity of AMPs, prompting the design of new analogs with powerful action than the parental peptides [28,29,30,31,32,33,34,35,36].

In the present study, a set of novel short peptides was designed based on previous research on the structural–functional relationship of AMPs and the identification of a key structural motif. The use of recognizing physicochemical parameters in combination with a template-modified strategy and the preliminary secondary structure analysis allowed the selection of a short novel peptide, named RKW (RKWILKWLRTWKK-NH2).

RKW includes 13 amino acids and forms a highly stable α-helical amphiphilic structure in the presence of model membranes. Biological investigation revealed that RKW exhibits a broad-spectrum and efficient bactericidal and antibiofilm activity against Gram-positive/Gram-negative pathogens, which include ESKAPE bacteria, and high fungicidal efficacy primarily against *Candida albicans*.

The valuable antimicrobial activity of the peptide and the low toxicity to normal mammalian cells should lay a good foundation for developing new potential RKW-based therapeutic or antiseptic agents.

## 2. Materials and Methods

### 2.1. Synthesis and In Silico Design of RKW

The peptide RKW investigated in this work was purchased from GenScript Biotech (Leiden, The Netherlands) in lyophilized form. As recommended by the manufacturer, RKW was stored at −20 °C. Before experimentation, fresh solutions in H_2_O or DMSO were prepared, briefly sonicated, and used as stock solutions in all analyses. The most important features of RKW such as the reliability, stability, half-life in vivo and all the relevant physicochemical properties (total net charge, hydropathicity, amphipathicity, hydrophobicity, Boman index, GRAVY index, aliphatic index, and instability index) were determined using the following web server and software: ProtParam (Expasy) (https://web.expasy.org/protparam/, accessed on 1 September 2024) [37], PlifePred (PPred) (https://webs.iiitd.edu.in/raghava/plifepred, accessed on 1 September 2024) [38], Antimicrobial Peptide Database3 (APD3) (https://aps.unmc.edu/, accessed on 1 September 2024) [39], and HeliQuest (https://heliquest.ipmc.cnrs.fr/index.html, accessed on 1 September 2024) [40]. The 3D structure of the peptide was predicted by AlphaFold3 (https://alphafoldserver.com/welcome, accessed on 1 September 2024) [41].

### 2.2. Circular Dichroism Spectroscopy

The secondary structure elements of the antimicrobial molecule were analyzed in a 0.1 cm optical path-length quartz cuvette (Hellma Analytics, Milan, Italy) using Jasco J-810 Circular Dichroism Spectropolarimeter (JASCO, Tokyo, Japan). All spectra were recorded at 25 °C in a wavelength range of 190–250 nm with a 1 nm bandwidth, a scanning speed of 20 nm/min, and averaged over five scans. The spectra as a function of SDS concentration (3, 50, and 150 mM) were obtained at 50 μM final concentration of peptide in 10 mM Tris-HCl buffer, pH 7.0. The folding kinetic analysis of the peptide was carried out after the addition of SDS (50 mM) to the sample (50 μM in 10 mM Tris-HCl, pH 7.0), up to 24 h incubation. The effect on conformational stability as a function of pH was examined by dissolving RKW at a concentration of 50 μM in 10 mM of different buffer solutions in a pH range 2.0–11.0 (glycine-HCl, pH 2.0; Tris-HCl, pH 7.0; glycine-NaOH, pH 11.0). Next, 50 mM of SDS was added to each sample, which was analyzed by CD spectroscopy after 48 h of incubation at 25 °C. The effect of temperature on the structural stability of the peptide was analyzed by dissolving the same concentration of RKW (50 μM) in 10 mM Tris-HCl, pH 7.0, kept at 4, 37, and 90 °C for 48 h before adding SDS at 50 mM concentration and acquiring the CD spectra. All CD spectra of RKW were corrected by subtracting a blank spectrum of a sample containing all components except the peptide (baseline correction). The mean residue ellipticity ([θ], deg. cm^2^ dmol^−1^) was obtained by the equation [θ] = 100 θ/cnl, where θ is the ellipticity (mdeg), c is the peptide concentration (mM), n is the number of residues, and l is the path length (cm).

### 2.3. Fluorescence Spectroscopy

Fluorescence experiments were conducted in a Shimadzu RF-6000 spectrofluorometer (Kyoto, Japan). The analyses were performed by setting an excitation and emission slit width of 5 nm and using a quartz cell with a path length of 1 cm. The intrinsic tryptophan residues were excited at a wavelength of 280 nm, following the emission between 300 and 400 nm. The effects of SDS on the peptide folding, the folding kinetics, and the thermal and the pH stability were monitored as described in Section 2.2.

### 2.4. Bacterial Strains

To assess the antimicrobial potential of the designed peptide, different strains of Gram-negative and Gram-positive bacteria were evaluated. *Pseudomonas aeruginosa* was an ATCC (27853) strain, while *Escherichia coli*, *Listeria monocytogenes*, *Staphylococcus aureus*, *Campylobacter jejuni*, *Salmonella* Typhimurium, the monophasic variant of *Salmonella* Typhimurium and *Salmonella* Napoli were isolated from food products by the Laboratory of Microbiological Food Control—Department of Food Microbiology of the Istituto Zooprofilattico Sperimentale del Mezzogiorno (IZSM) in Portici (Naples, Italy).

### 2.5. Antimicrobial Assays

In accordance with the Clinical & Laboratory Standards Institute guidelines (CLSI, 2015), the Minimum Bactericidal Concentration (MBC), defined as the minimum concentration of peptide required to kill 99.9% of the tested bacterial suspension within a defined time interval, was determined. *L. monocytogenes*, *E. coli*, *S. aureus*, S. Typhimurium, monophasic variant of *S.* Typhimurium, *S.* Napoli, and *P. aeruginosa* were cultured at 37 °C in buffered peptone water (Thermo Fisher, Milan, Italy), while *C. jejuni* was cultured in Bolton Broth (Oxoid, Madrid, Spain). The bacterial suspension was collected and then diluted in fresh broth to a final concentration of 1.0 × 10^3^ or 1.0 × 10^5^ colony-forming units (CFUs)/mL. Different concentrations of RKW (ranging from 1 to 100 µM) were prepared by diluting the peptide in the suitable medium starting from a stock solution in DMSO and added to each bacterial suspension, following incubation at 37 °C for 6 h. Samples containing only cell suspension and DMSO were used as controls. Therefore, 50 μL of each bacterial cell suspension was transferred onto selective agar plates (*L. monocytogenes*, Agar Listeria acc. to Ottaviani & Agosti (ALOA)—Biolife Italiana; *S*. Typhimurium, monophasic variant of *S*. Typhimurium and *S*. Napoli, Salmonella Chromogenic agar—Oxoid UK; *S. aureus*, Baird Parker agar base—Biolife Italiana; *E. coli*, TBX agar—Biolife Italiana; *C. jejuni*, modified Charcoal Cefoperazone Deoxycholate Agar-Liofilchem, Roseto degli Abruzzi, Italy; *P. aeruginosa*, pseudomonas agar base with CFC supplement-Oxoid, Madrid, Spain) and incubated 24/48 h at 37 °C for *L. monocytogenes* (ISO 11290-1:2017), *S*. Typhimurium (ISO 6579-1:2020), monophasic variant of *S*. Typhimurium (ISO 6579-1:2020), *S*. Napoli (ISO 6579-1:2020), and *S. aureus* (ISO 6888-1:1999) while *E.coli* (ISO 16649-1:2018) and *C. jejuni* (ISO 10272-2:2017) were incubated 24 h at 44 °C, and 48 h at 41 °C, respectively. P. aeruginosa was incubated at 25 °C for 48 h (ISO 13720:2010). Data are the average of three independent experiments; each performed in triplicate.

### 2.6. Antimicrobial Resistance of ESKAPE Bacteria

The antimicrobial susceptibility of the four ESKAPE bacteria *Acinetobacter baumannii*, *Enterococcus faecium*, *P. aeruginosa*, and *S. aureus*, made available by the culture collection of IZSM, was evaluated by the disk diffusion method. Antimicrobials used were different and specific per each bacterium under study. Specifically, for *A. baumannii*, the susceptibility was evaluated against amikacin (30 μg), gentamicin (10 μg), tobramycin (10 μg), imipenem (10 μg), meropenem (10 μg), ciprofloxacin (5 μg), levofloxacin (5 μg), and trimethoprim-sulfamethoxazole (1.25–23.75 μg); *E. faecium* was tested towards imipenem (10 μg), ciprofloxacin (5 μg), levofloxacin (5 μg), vancomycin (5 μg) and ampicillin (2 μg); *P. aeruginosa* versus amikacin (30 μg), imipenem (10 μg), meropenem (10 μg) and tobramycin (10 μg); finally, for *S. aureus* were selected amikacin (30 μg), gentamicin (10 μg), tobramycin (10 μg), cefoxitin (30 μg), ciprofloxacin (5 μg), levofloxacin (5 μg), erythromycin (15 μg), tetracycline (30 μg) and trimethoprim-sulfamethoxazole (1.25–23.75 μg). Thereby, the diameter of the inhibition zones was measured, and bacteria were classified as resistant or sensitive per antibiotic tested in agreement with the European Committee for Antimicrobial Susceptibility Testing (EUCAST, 2025) breakpoints [42].

### 2.7. Antimicrobial and Antibiofilm Activity Against ESKAPE Bacteria

The antimicrobial activity of RKW against four ESKAPE bacteria was assessed by the broth microdilution method performed in 96-well microplates according to EUCAST 2025 [42] and ISO 20776-1 guidelines. Therefore, the minimum inhibitory concentration (MIC) and MBC were evaluated. The applied protocols followed those proposed by Gratino et al. [43], testing different concentrations of RKW (from 1 to 75 μM) against bacterial inocula of 3 and 5 Log (CFU/mL) for each microorganism. Subsequently, the antibiofilm activity of the peptide was tested according to Festa et al. [34]. Specifically, two different concentrations of peptide were used, corresponding to ½ and ¼ of the estimated MIC value, which guarantee the optimal bacterial growth conditions necessary to allow biofilm production. Biofilm production (%) was calculated based on Optical Density (measured at 630 nm) outputs, compared to the control group.

### 2.8. In Vitro Cytotoxicity Assays

The toxicity of RKW on mouse embryo fibroblasts BALB 3T3 clone A31 (ATCC CCL-163) was determined using the Neutral Red Uptake (NRU) assay, according to ISO 10993-5:2009. BALB 3T3 clone A31 (ATCC CCL-163) cells were cultivated in Dulbecco’s Modified Eagle’s Medium (D-MEM) supplemented with 10% Hi-NCS (Heat inactivated at 57 °C for 45 min-Newborn Calf Serum) and 4 mM Glutamine. For the NRU assay, the cells were seeded in a 96-well microtiter plate (Thermo Fisher Scientific, Milan, Italy) and incubated for 24 h at 37 °C and 5% CO_2_ in a humidified environment, allowing cell sedimentation and the constitution of a sub-confluent monolayer before treatment with RKW. After 24 h, the 96-well plate was removed from the incubator, and the growth medium was discarded. Therefore, cells were incubated with increasing concentrations of RKW (5, 10, and 25 µM) prepared in treatment medium (DMEM with 5% Hi-NCS, 4 mM Glutamine, 0.1% DMSO) for 24 h at 37 °C, using the treatment medium as a negative control and a solution of SDS (0.5 mg/mL) prepared in the treatment medium as a positive control. Next, each well was rinsed with 150 µL of D-PBS with Ca^2+/^Mg^2+^ and incubated with 100 µL Neutral Red (NR) dye solution (50 µg/mL) for 3 h at 37 °C. Afterwards, each well was again rinsed with 150 µL of D-PBS with Ca^2+/^Mg^2+^, and 150 µL of NR desorb solution (49% ddH_2_O, 50% ethanol, 1% acetic acid) was added. Plates were placed under gentle agitation in the dark for 10 min, and the Optical Density of the NR extract at 540 nm was measured spectrophotometrically. Cell viability was expressed as a percentage of BALB 3T3 clone A31 cells grown in the treatment medium (DMEM with 5% NCS, 4 mM Glutamine, 0.1% DMSO, and RKW), respect to the control group represented by the cells grown in DMEM with 5% NCS, 4 mM Glutamine, and 0.1% DMSO (viability control cells = 100%).

(1) (OD treated cells − OD blank)/(OD Control Cells − OD blank) × 100

Interpretation of the data was carried out according to ISO 10993-5:2009: the compound was considered cytotoxic if the relative cell viability of the sample was <70% of the control group, while it was considered non-cytotoxic if the cell viability of the sample was ≥70% of the control group.

### 2.9. In Vitro Evaluation of the Stability of RKW in Newborn Calf Serum

A total of 3.7 mg of peptide was dissolved in 1 mL of Milli-Q H_2_O to obtain a 2 mM stock solution. A 5% solution of Newborn Calf Serum (NCS) was prepared in Dulbecco’s phosphate-buffered saline (DPBS) (*v*/*v*) and then subjected to heat-inactivation by incubating the solution at 57 °C for 45 min (Hi-NCS). Therefore, 100 µM of peptide was incubated in 5% Hi-NCS at 37 °C for 24 h in DPBS. At two different times (0 and 24 h), 100 µL of each solution was recovered and mixed in a 1:1 ratio (*v*/*v*) with acetonitrile and 0.1% TFA to stop further degradation of RKW and precipitate serum proteins. Therefore, the samples were centrifuged at 13,000 rpm for 5 min, and the supernatants were diluted in an equal volume of water. Finally, 200 µL of the collected solutions were loaded onto a μBondapak reversed-phase C18 column (Waters, 3.9 mm × 300 mm) connected to an HPLC system (Shimadzu, Milan, Italy), using a linear gradient of acetonitrile (5–95%) in water containing 0.1% TFA at a flow rate of 1 mL/min, and absorbance was detected at 280 nm. The peptide stability was determined by comparing the peak area of RKW incubated for 24 h at 37 °C in the presence of Hi-NCS with that at time 0. Samples containing only Hi-NCS were also analyzed. The same experiments were performed in the presence of NCS, not subjected to the heat inactivation treatment. Results are the average of three independent studies.

### 2.10. In Vitro Evaluation of Bactericidal and Fungicidal Activity of RKW According to European Standard Guidelines

The method is based on the suspension test according to BS EN 1276:2019, which specifies a quantitative suspension analysis for the evaluation of the bactericidal activity of chemical disinfectants and antiseptics used in food, industrial, domestic, and institutional areas against a panel of microorganisms under conditions that more closely simulate practical use. Briefly, *S. aureus* ATCC 6538, *P. aeruginosa* ATCC 15442, and *Staphylococcus epidermidis* ATCC 12228 were used as test organisms at a bacterial concentration of 1.0 × 10^8^ colony-forming units (CFU) per mL (CFU/mL). In the test, BSA (Bovine Serum Albumin) (0.3 g/L) was used as an interfering substance to mimic clean conditions. The mixture formed by the interfering substance and the bacterial suspension was homogenized and kept at 37 °C, followed by the addition of RKW at the desired concentration (ranging from 5 to 100 µM). The reaction mixture was incubated at 37 °C for 60 min before transferring each sample to a tube containing a neutralizer solution consisting of 30 g/L polysorbate 80, 1 g/L sodium thiosulfate, 3 g/L lecithin, 1 g/L tryptone, 8.5 g/L NaCl, 100 mL/L phosphate buffer and water. The negative control (untreated cells) was prepared using PBS instead of biocides. The solutions were then plated onto Tryptone Soy Agar (TSA) and incubated at 37 °C for 48 h to determine the logarithmic CFU/mL reduction, according to the following Equation:

(2) log R = log (N_0_) − log (N_a_)

where R refers to the reduction in viability, N refers to the number of CFU/mL in the test mixture at the beginning of the contact time, and N_a_ refers to the number of CFU/mL in the test mixture at the end of the contact time. The compound meets the requirements of the EU when, after 60 min of contact time of the product and bacterial suspension at the appropriate temperature, it demonstrates against bacteria at least a decimal log reduction in counts of 5 (reduction ≥5 log CFU/mL). The test method and the minimum requirements for fungicidal activity of chemical disinfectant and antiseptic products were performed according to BS EN 1650:2019. The fungicidal activity was studied using the vegetative cells of *Candida albicans* ATCC 10231 and the spores of *Aspergillus brasiliensis* ATCC 16404 as test organisms. Briefly, RKW at the desired concentrations (ranging from 5 to 100 µM) was added to a test suspension of fungi (1.0 × 10^6^ CFU/mL) under simulated clean conditions (0.3 g/L bovine albumin solution). The mixture was maintained at 37 °C for a contact time of 60 min. At the end of this contact time, an aliquot was taken, and the fungicidal activity was immediately neutralized with a solution consisting of 30 g/L polysorbate 80, 1 g/L sodium thiosulfate, 3 g/L lecithin, 1 g/L tryptone, 8.5 g/L NaCl, 100 mL/L phosphate buffer and water. The samples were then plated onto malt extract agar (MEA) and incubated at 30 °C for 48 h to determine the numbers of surviving fungi as the logarithmic CFU/mL reduction, calculated according to Equation (2). The compound meets the requirements of the EU when, after 60 min of contact time, it demonstrates against fungi at least a decimal log reduction in counts 4 (reduction ≥4 log CFU/mL).

### 2.11. Statistical Analysis

To compare the percentages of biofilm production across different samples, a one-way analysis of variance (ANOVA) was conducted using Prism v. 10.0 (GraphPad, Graph-Pad Software, La Jolla, CA, USA). Significant differences between sample pairs were identified with Tukey’s post hoc test (*p* < 0.05).

## 3. Results and Discussion

### 3.1. Design of a New Lysine-Tryptophan-Rich Peptide

Antimicrobial peptides (AMPs), belonging to the innate immune system of animals and plants, have emerged as a promising option for new antibiotic treatments [44,45]. These amphiphilic compounds show a broad spectrum of activity against different pathogens and the ability to bind to the surface of the bacterial membranes via electrostatic interactions, leading to structural destabilization and cell death [22,23]. In contrast to conventional antibiotics, this approach makes it difficult for bacteria to develop antibiotic resistance, which is one of the top global public health and development threats [46,47]. Therefore, naturally occurring AMPs, their mutants, and synthetic peptides are intensively screened and designed to be used as new types of therapeutic agents to fight bacterial infections [26,48]. Unfortunately, the scarce stability under different environmental conditions, the low efficacy against severe infections caused by diverse pathogens, the potential toxicity, and the high production costs has strongly limited the therapeutic applications of the first-generation of antimicrobial peptides [35,49,50].

Therefore, starting from the sequences of the previously characterized antimicrobial peptides [28,29,30,31,32,33,34], a panel of more effective and stable AMPs was designed. The adopted criteria followed the typical chemical and physical properties, which influence AMPs interaction with the negatively charged cell envelope and their tendency to assume amphipathic conformations upon binding to the bacterial membrane, such as cationicity, hydrophobicity, amphipathicity, and the spatial arrangements of the side chains. In addition, the projected strategy has been driven by the identification of a further key structural feature related to de novo motif “WX_1_X_2_X_3_W” (in which X_2_ or X_3_ is R or K), showing two Trp residues located at a proper distance for mutual stacking and helix stabilization. This hydrophobic bunch “WXXXW”, when repeated twice, as in the cationic AMP RILK1, resulted in an improvement ability of the peptide to adopt amphipathic structures stabilized on one face by the presence of a Tryptophan cluster, and on the other by the interaction between the positively charged amino acids and the negatively charged target membranes [51]. Indeed, Trp is a residue often present in high proportion in AMPs as it significantly contributes to their antimicrobial activity [52,53]. The uniqueness of this hydrophobic amino acid derives from the side chain indolic ring, allowing AMPs to anchor to the bacterial membrane due to its binuclear aromatic structure, which affects the interface region of lipid bilayer and disturbs the internal structure of cells [43,53]. However, it has also been observed that while the occurrence of several Trp in AMP sequences plays a crucial role in their selectivity, guaranteeing efficient antimicrobial activity, the presence of four or more Trp residues could increase their cytotoxicity [54]. In essence, all these crucial properties work “in concert” and their combination can govern how AMPs interact with microbial membranes, dictating their antimicrobial potency and selectivity, and therefore their potential as therapeutic agents. Specifically, a higher net cationic charge may enhance initial membrane binding as electrostatic attraction is a primary step in the peptide’s mechanism of action, but a sufficient hydrophobicity and a favorable hydrophobic moment together with a good proportion of Trp content (more than 20%) are fundamental for the efficient insertion of AMPs and membrane disruption.

Therefore, the new set of AMPs was rationally designed by including multiple Trp-based motifs such as “WXXXW” along the peptide chains. In detail, the projected strategy has involved the combined and integrated use of different software, such as ProtParam [37], PPred [38], APD3 [39], and HeliQuest [40], among those most frequently applied for in silico analysis of peptide sequences. This approach enables accurate determination of the physicochemical properties, which are important for the adopted predictive process and generally associated with the peptide’s biological function. Therefore, a 13-mer peptide, named RKW, was designed and selected, following the construction of an ad hoc decision-tree model, which was applied starting from the sequences of the new and already known panel of AMPs. Specifically, the developed model comprises a series of “decision nodes”, each representing a “test” on one of the ten identified physicochemical parameters (half-life, hydrophobicity, hydropathicity, amphipathicity, hydrophilicity, net charge, Boman index, hydrophobic moment, secondary structure, sequence length). To choose among alternatives and optimize the decision quality, criteria such as threshold limits, were used to evaluate and identify those candidates that led to the definition of the best promising peptide of this study in terms of potential antimicrobial activity, high stability, non-cytotoxicity, and small size. Thus, RKW was chosen for further biophysical and in vitro antimicrobial testing.

Indeed, RKW displayed an improvement of the instability index, which is critical for therapeutic applications, and some interconnected chemico-physical features such as the net cationic charge, whole-residue hydrophobicity, total Trp-ratio, and the hydrophobic moment “μH” (Table 1), compared to the previously characterized AMPs [28,29,30,31,32,33,34].

### 3.2. Peptide Molecular Modeling

The insertion of helical peptides into cell membranes is an enthalpy-driven process, stabilized by helix formation and nonclassical hydrophobic interactions between apolar side chains and the lipid bilayer, leading to membrane reorganization.

Cholesterol’s protective role in eukaryotic membranes against AMPs may be linked to its influence on this lipid remodeling. As the energetic barrier necessary to introduce peptides into the bilayer is reduced by hydrogen bond formation, extensive helix formation is encouraged, even in shorter antimicrobial peptides. These factors underscore that the ability to form a well-defined, amphipathic α-helix is a strong determinant of antimicrobial activity. Hence, the potential of a peptide to adopt α-helical conformations upon encountering a membrane-like environment, rather than its intrinsic helical stability, could be the key factor in its antimicrobial potency [35,48].

**Figure 1:**
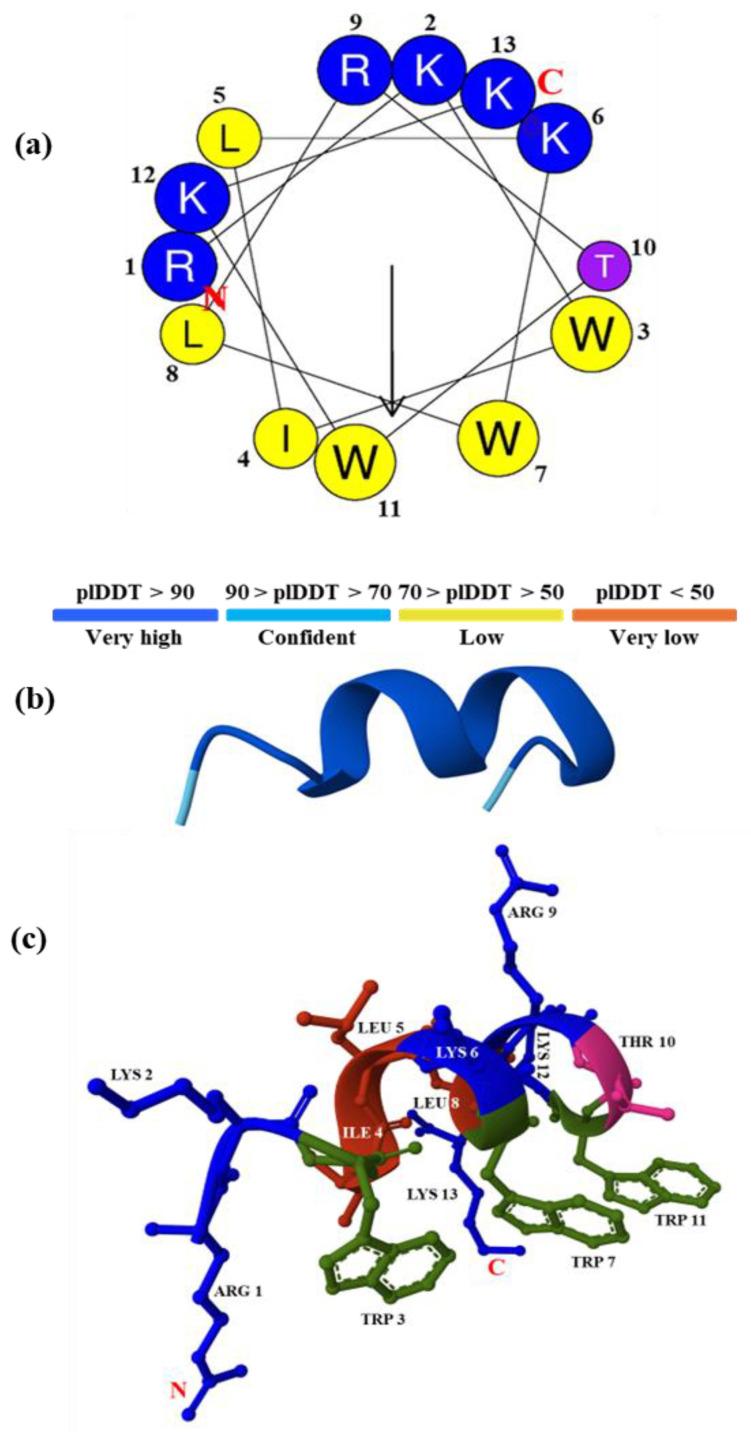
Helical wheel projections and molecular structure of the rationally designed peptide RKW. (**a**) Helical wheel projections of RKW obtained with the HeliQuest software. Hydrophobic amino acids are indicated as yellow circles, and hydrophilic amino acids are shown as blue circles. The hydrophobic moment is represented by a black arrow on the helical wheel, and the letters N and C in red indicate the N- and C-terminals, respectively; (**b**) 3D structure of RKW predicted by AlphaFold3 represented in cartoon with a color-coded score. pLDDT: predicted local distance difference test; (**c**) 3D structure of RKW predicted by AlphaFold3 illustrating the α-helical structure of the peptide. Side chains are represented in ball-and-stick, while the backbone in cartoon. N- and C-terminal residues were indicated in red.

Herein, the conformation of the modeled peptide RKW was built to be an α-helix with distinct amphiphilic surfaces. As depicted in Figure 1, the polar pattern is generated by the positively charged Lys and Arg and the hydrophilic Thr residues. The apolar surface of the α-helix is mainly composed of the three aromatic Trp residues in positions 3, 7, 11, and the hydrophobic cluster of Ile, Leu, and Leu in positions 4, 5, and 8, which makes the corresponding molecular surface much more pronounced and larger in size. Therefore, the rational peptide design has led to the discovery of an amphipathic α-helix with potential antimicrobial properties by adjusting the distribution and composition of the amino acid residues starting from peptide templates. Moreover, the predicted α-helical model has very high confidence levels (pLDDT > 90), confirming the high accuracy of our results.

### 3.3. Spectroscopic Characterization

Several studies have indicated that AMPs undergo conformational changes upon binding with bacterial membranes, assuming a well-defined structure that represents an important requirement to penetrate the membrane and exert their antimicrobial activity [55,56].

**Figure 2:**
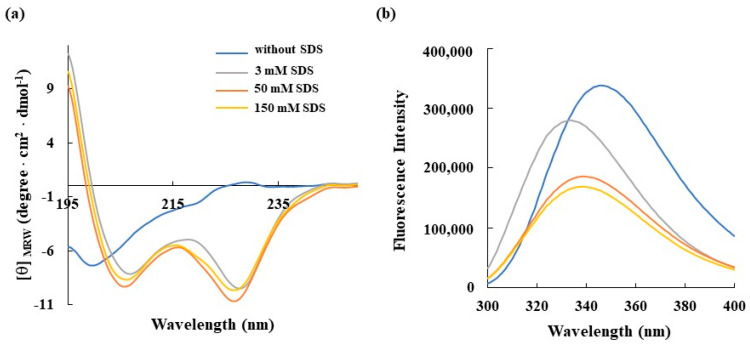
Effect of SDS concentration on the secondary structure and conformation of RKW monitored by spectroscopic techniques. (**a**) CD spectra and (**b**) fluorescence emission spectra of RKW. All spectra were recorded at 25 °C using a peptide concentration of 50 μM in 10 mM Tris-HCl buffer, pH 7.0, and in the absence (orange lines) or in the presence of SDS at different concentrations.

In this context, fluorescence and CD experiments were performed to study conformational and secondary structural changes in RKW in the presence of membrane-mimicking agents. To this aim, the detergent SDS was chosen for the spectroscopic analyses as it is negatively charged, making it a good model to mimic bacterial membranes. In a first set of experiments, SDS was titrated against the peptide at a final concentration of 50 μM to evaluate the secondary structure formation upon interaction. Far-UV CD spectra (Figure 2a) showed that RKW was unstructured in buffer solution as evidenced by the negative band close to 200 nm, but the addition of SDS at 3 mM concentration induced a coil to α-helix transition, evidenced by the presence of the negative double-minimum peaks at 208 and 222 nm and a maximum at 190 nm. This behavior indicates that RKW is prone to assuming a highly ordered conformation when interacting with membrane-mimicking agents like SDS, and specifically, the α-helix formation permits an efficient permeabilization of the hydrophobic core of bacterial membranes [24].

Moreover, the shapes and intensities of the CD spectra remained similar upon increasing of SDS concentration above the critical micelle concentration (CMC), meaning that, once adopted, the α-helical structure does not further depend on the SDS concentration. To obtain more information on the conformational changes in RKW when interacting with a membrane-mimetic system, fluorescence spectroscopy was employed, considering that the fluorescence of tryptophan (W) residues is strongly influenced by the polarity of the surrounding microenvironment. As depicted in Figure 2b, the emission maximum of RKW in aqueous solution was ∼350 nm, which is typical for Trp in a polar environment, whereas upon addition of SDS at concentrations both lower (3 mM) and higher (50 and 150 mM) than that of micellar one, a blue shift in the λmax and a marked decrement in the emission intensity occurred, indicating that the dynamic property of the Trp residues is limited and they are buried in the hydrophobic environment of the SDS, resulting in their shielding.

**Figure 3:**
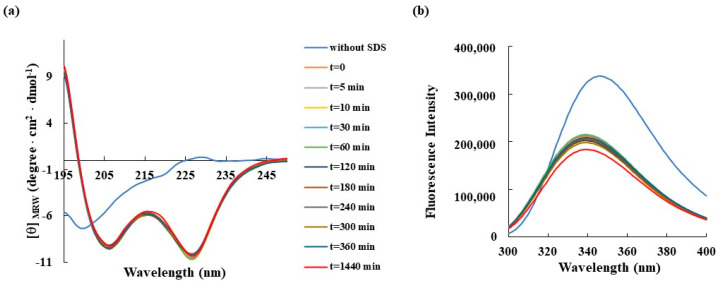
Time-dependent effect of SDS on the secondary structure and conformation of RKW monitored by spectroscopic techniques. (**a**) CD spectra and (**b**) fluorescence emission spectra of RKW. All spectra were recorded at a peptide concentration of 50 μM in 10 mM Tris-HCl, pH 7.0, in the absence (blue lines) or in the presence of SDS (50 mM) up to 24 h incubation at 25 °C.

Then, the conformational rearrangements of RKW in the presence of 50 mM SDS were studied over 24 h period, during which the dichroic (Figure 3a) and fluorescence (Figure 3b) spectra were recorded at specific time points. The results obtained from both Circular Dichroism and fluorescence analyses demonstrated that the peptide did not markedly change its α-helical conformation during 24 h incubation once it had been adopted (t = 0).

**Figure 4:**
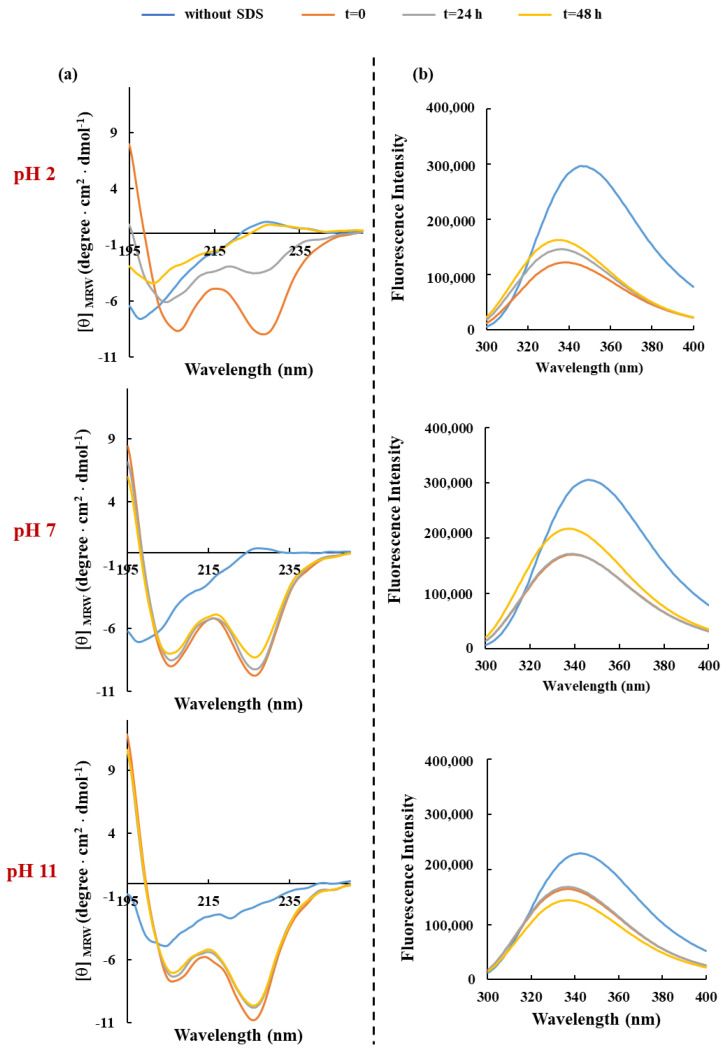
Effect of pH on the secondary structure and conformation of RKW. (**a**) CD spectra and (**b**) fluorescence emission spectra of RKW. All spectra were obtained by incubating the peptide (50 μM) in buffers at different pHs for 48 h at 25 °C, and in the presence of SDS at a final concentration of 50 mM.

**Figure 5:**
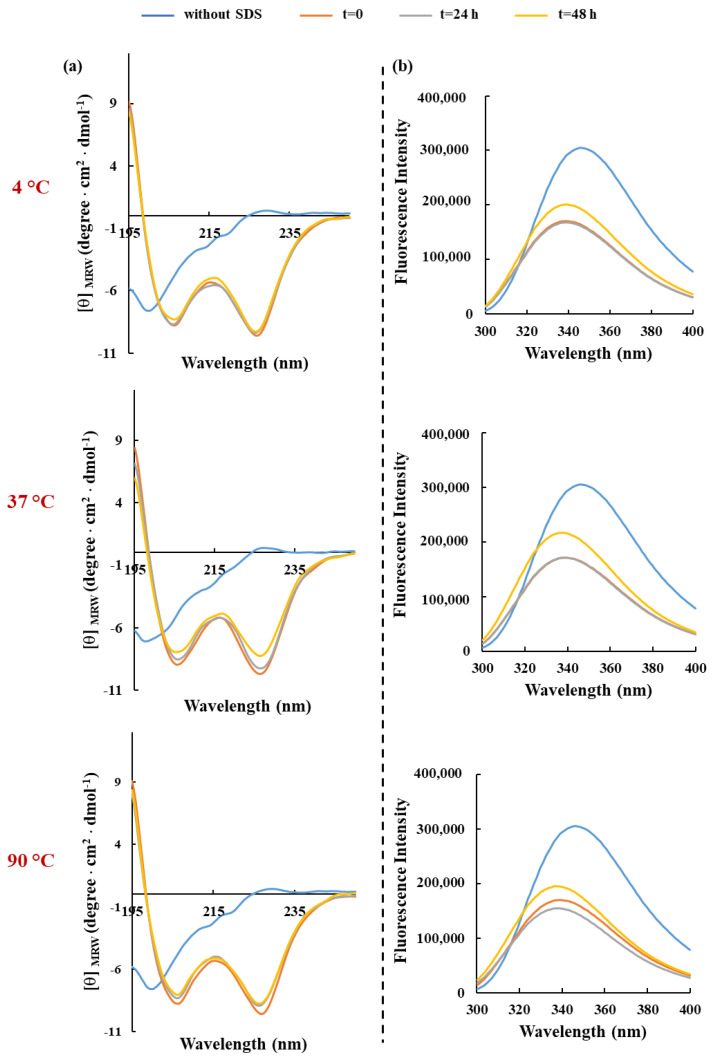
Effect of the temperature on the secondary structure and conformation of RKW. (**a**) CD spectra and (**b**) fluorescence emission spectra of RKW. All spectra were acquired by incubating the peptide (50 μM) in 10 mM Tris-HCl buffer, pH 7.0, in the presence of 50 mM SDS at three different temperatures up to 48 h of incubation.

One of the many limitations of developing AMPs for clinical or industrial applications is their low stability in certain environments, which significantly reduces their antimicrobial potency and efficacy. In this context, the pH and the thermal stability represent critical physicochemical conditions for their preparation, processing, and storage. Hence, the effects of both parameters on peptide-SDS complexes were investigated within 48 h of incubation. Our results demonstrated that only in extreme acidic conditions (pH 2.0) does the helical content of RKW gradually decrease during the time, becoming entirely disordered over 48 h of incubation. Conversely, the structural (Figure 4a and Figure 5a) and folding stability (Figure 4b and Figure 5b) of RKW when in complex with SDS micellar solutions, were not markedly affected under all other pH and temperature conditions considered, thus confirming the ability of molecules to adapt to different environments and its high stability.

### 3.4. In Vitro Evaluation of Antibacterial Activity of RKW

To evaluate the bactericidal potential of the designed peptide, MBC determination was performed in vitro by the broth microdilution method against eight bacterial strains commonly associated with human infections, including two Gram-positive (*Staphylococcus aureus* and *Listeria monocytogenes*), and six Gram-negative (*Escherichia coli*, *Pseudomonas aeruginosa*, *Salmonella* Typhimurium, *Salmonella* Napoli, monophasic variant of *Salmonella* Typhimurium, and *Campylobacter jejuni*) bacteria.

In an initial screening, the bactericidal effect of RKW was tested by incubating 1.0 × 10^3^ CFU/mL of pathogens with increasing peptide concentrations ranging from 1 to 100 µM for 6 h at 37 °C. As summarized in Table 2, RKW was extremely effective against the selected Gram-positive as well as Gram-negative bacterial strains, with the most potent bactericidal activity exhibited at low doses against *P. aeruginosa*, one of the most common pathogens that is resistant to most antibiotics currently in use and causes a variety of severe infection diseases especially in the hospital environments. RKW was less efficient against *C. jejuni* strain, affecting cell viability only at high concentration (80 μM).

As the activity and consequently the efficacy of many antibiotics depends on the bacterial load that vary by several orders of magnitude in relevant infections, a second set of experiments was conducted using a higher initial cell concentration equal to 1.0 × 10^5^ CFU/mL (a value considered indicative of a significant infection) of the strains resulted previously more susceptible to the peptide’s action (Table 3). Representative plates of RKW against the tested pathogens were reported in Figure S1.

The results obtained were much more impressive, confirming the important antimicrobial activity exhibited at low concentrations by RKW against the studied strains, and specifically against the Gram-negative bacteria such as *P. aeruginosa*. Indeed, this group of pathogens, due to the complex composition of their envelope, limits the entry of antibiotics into the cell, thus rendering the related infections difficult to treat [55,56,57].

Therefore, the identification of new small molecules that can be active against this class of pathogens remains a challenge, which has created delays in developing new AMPs to deal with them. Taken together, these data provide some insights into the possibility that the projected peptide could represent a promising option and an alternative to traditional antibiotics for the treatment of severe infections caused by both classes of pathogens.

### 3.5. Peptide Investigation to Address Antimicrobial Resistance and Tolerance of ESKAPE Bacteria

Antibiotic resistance, as well as antibiotic tolerance, strongly influences the rate of therapeutic failures [58]. Nowadays, for a molecule to be considered a promising anti-biotic candidate, it has to offer benefits in combating multidrug-resistant pathogens (MDR) such as ESKAPE bacteria, which are included by the World Health Organization (WHO) in the list of high and critical priority pathogens [59]. Specifically, the critical-priority group includes the Gram-negative ESKAPE bacteria such as *Klebsiella pneumoniae*, *Escherichia coli*, *Enterobacter* species, both those resistant to carbapenem (*Enterobacterales*, carbapenem-resistant) and those resistant to third-generation cephalosporin (*Enterobacteriales*, third-generation cephalosporin-resistant), and *Acinetobacter baumannii* carbapenem-resistant (CRAB). Within the “high group” of priority pathogens are both the Gram-positive ESKAPE pathogens, such as *Enterococcus faecium* vancomycin-resistant and *Staphylococcus aureus* methicillin-resistant, and the Gram-negative pathogen *Pseudomonas aeruginosa* carbapenem-resistant [60].

In this scenario, to address the problem of therapeutic failures related to antibiotic resistance and tolerance, the novel and promising antimicrobial peptide RKW was also tested against ESKAPE microorganisms. Firstly, the phenotypic approach was used to evaluate their antimicrobial resistance profile, and the results of disk diffusion tests were analyzed according to EUCAST guidelines [42]. The antimicrobial resistance profiles of ESKAPE strains were reported in Table 4.

In detail, while the *P. aeruginosa* strain did not show resistance to any of the antibiotics tested, *A. baumannii*, *E. faecium*, and *S. aureus* exhibited resistance to more than two classes of antibiotics and are, therefore, classified as MDR strains (Table 4). Notably, *A. baumannii* was not susceptible to any of the eight antibiotics tested, not even to carbapenem molecules (Table 4). These findings are particularly relevant as they place this strain in the WHO’S critical priority category, which is characterized by limited therapeutic options, high disease burden (mortality and morbidity) and global resistance mechanisms. Also challenging to treat are the bacteria belonging to the high priority category, which are critical for certain populations and specific areas based on their “geographic impact” [60]. This group includes bacteria like *E. faecium* vancomycin-resistant and *S. aureus* methicillin-resistant, just like those included in the present study. The *E. faecium* strain tested was resistant to Ciprofloxacin, Levofloxacin, Ampicillin, and Vancomycin (Table 4). This pathogen is responsible for highly feared infections, often due to multidrug-resistant bacteria, causing severe and complex pathological manifestations that can lead to death (high morbidity and mortality) [61]. *E. faecium* is considered an MDR bacterium due to its intrinsic resistance to aminoglycosides such as tobramycin, kanamycin, and gentamicin. However, the acquired resistance to Vancomycin (Vancomycin-Resistant *Enterococci* (VRE); VRE*fm* strains) is most concerning and is frequently reported in episodes of serious infections in hospital settings [61]. Regarding the *S. aureus* strain, despite some reported antibiotic susceptibility (Table 4), important resistance was detected against four of the selected antibiotics: Ciprofloxacin, Levofloxacin, Erythromycin, and Cefoxitin, a β-lactam molecule typically used for the detection of Methicillin-resistant strain (MRSA, Methicillin-resistant *S. aureus*) [62]. Methicillin resistance, defined by the expression of penicillin-binding protein 2a (PBP2a), renders Methicillin and other β-lactam antibiotics ineffective against MRSA strains. It is significant that the WHO considers *S. aureus* a serious threat to human health worldwide due to its pathogenicity and resistance to antibiotics [63,64].

Once the antimicrobial resistance profile of ESKAPE strains was characterized, the antimicrobial peptide RKW was tested against them, taking up the challenge of also verifying its antimicrobial efficacy against difficult-to-treat bacteria, such as CRAB, VRE*fm*, and MRSA. This goal has been achieved by incubating different concentrations of the peptide with bacterial monoculture at concentrations of 3 or 5 Log (CFU/mL). Specifically, the *A. baumannii* carbapenem-resistant strain was found to be susceptible to RKW peptide when used at concentrations between 50 and 75 μM. Our findings suggest a direct relationship between the concentration of the bacterial inoculum and the dosage of peptide required to exert both bacteriostatic and bactericidal effects on microorganisms. Indeed, the minimum concentration of RKW exhibiting bactericidal activity was influenced by the initial bacterial load. Consequently, the MBC was set at 75 μM for an inoculum of 3 Log (CFU/mL) and above 75 μM for an inoculum of 5 Log (CFU/mL) (Table 5). Similar considerations could be made regarding the *P. aeruginosa* strain, for which the effective concentration of the peptide must be modulated based on the bacterial concentration. Conversely, the effective peptide concentrations for the other two bacteria appeared consistent across both inoculum concentrations tested. In detail, the *E. faecium* Vancomycin-resistant strain was inhibited in growth by RKW at 20 μM, a concentration effective even when the bacterial inoculum concentration was around 5 Log (CFU/mL). However, a difference in MBC values was recorded when comparing the results obtained with the two inoculum concentrations (Table 5). Similarly, the MRSA strain proved to be sensitive to RKW activity with an estimated MIC value of 50 μM, regardless of the inoculum concentration (3 or 5 Log (CFU/mL)). Interestingly, in this case, the MIC values overlapped with those of MBC (Table 5). These results open interesting and promising horizons in the treatment of severe and insidious infections caused by MDR pathogens. The peptide RKW boasts antimicrobial activity at low concentrations against MDR bacteria isolated in intensive care units, where ESKAPE pathogens are the main cause of mortality and morbidity [65]. These encouraging data could give hope to the scientific community and international organizations, in the continuous research of novel molecules that can replace those commonly used. The use of antimicrobial peptides, such as RKW, could help alleviate the pressure of the phenomenon of antimicrobial resistance on therapeutic choices, which now must follow precise operational standards to reduce therapeutic failures.

**Figure 6:**
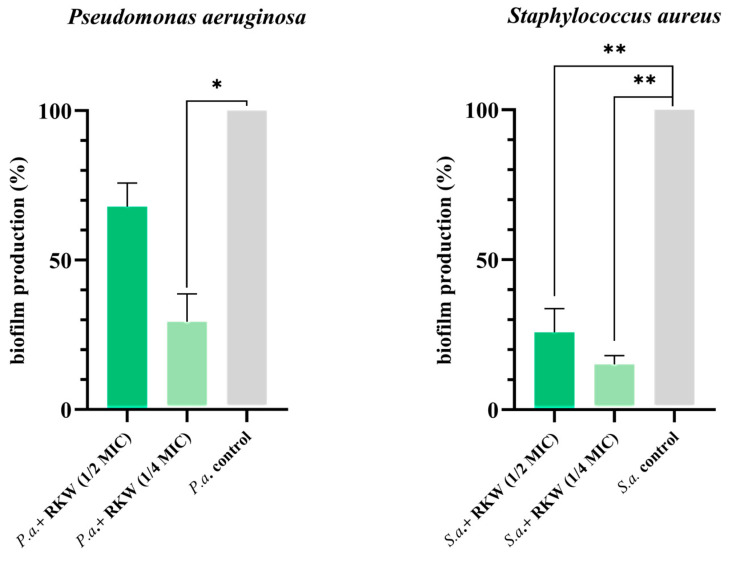
Inhibitory effect of RKW on biofilm formation assessed for *S. aureus* (MRSA) and *P. aeruginosa* strains. Specifically, 25 and 12.5 μM of RKW were used as ½ and ¼ MIC for *S. aureus* (*S.a*.), while 10 and 5 μM were used for *P. aeruginosa* (*P.a*.). Results are shown as the mean expression fold (± standard error of the mean) from three independent experiments. Statistical significance was assessed using a parametric one-way ANOVA, and significant differences are indicated on the histogram bars (* *p* < 0.05, ** *p* < 0.01).

In addition to resistance, bacteria can survive antimicrobial treatments through tolerance. This occurs when bacterial growth is inhibited but resumes after drug exposure ends [58]. Tolerance is often related to reduced activity of the antimicrobial target and is, therefore, influenced by the physiological state of the cell. Slow or no-growth states, such as the prolonged lag phase, stationary phase, biofilms, persister cells, and small-colony variants, allow bacteria to evade the lethal action of antimicrobials. In biofilms, bacterial communities enveloped in an extracellular matrix, a significant portion of the population exhibits slow or no-growth, a mechanism that is thought to promote tolerance [66]. For this reason, the antibiofilm properties of peptide RKW were evaluated by using the same ESKAPE bacteria; however, CRAB and VRE*fm* strains were not biofilm producers. As shown in Figure 6, RKW significantly inhibited the biofilm production by both *S. aureus* MRSA and *P. aeruginosa* (ANOVA, *p* value < 0.01 and <0.05, respectively), even when used at concentrations of one-half or one-quarter of the minimum inhibitory dose (½ and ¼ MIC). Interestingly, the lowest concentration of peptide tested (¼ MIC: 12.5 μM and 5 μM per *S. aureus* MRSA and *P. aeruginosa*, respectively) caused the greatest inhibition of biofilm production (Figure 6). Considering that many nosocomial infections are caused by bacteria that persist in the environment thanks to their ability to produce biofilms, the use of molecules with antibiofilm activity would allow us to address the problem of antimicrobial tolerance. To fully grasp the underlying mechanisms of the antibiofilm activities, further analyses are essential. Indeed, the current method, although useful, does not offer enough insight into specific details like the viability of cells within the extracellular matrix or the exact mechanisms of action. However, the crystal violet assay effectively demonstrates the potent antibiofilm capabilities of the peptide and its potential application against sessile bacterial cells, which represent a serious issue across diverse environments.

Therefore, based on the results of the activity assays of the peptide against ESKAPE bacteria, including critical and high-priority ones, it could be reasonably hypothesized that in the post-antibiotic era, the RWK peptide could enter the list of candidate molecules for the therapy of difficult-to-treat infections.

However, to validate and expand the antibiofilm action of RKW, further studies will be conducted to assess its effectiveness against a wider range of bacterial species than those currently examined, including important ones like *Klebsiella pneumoniae*.

### 3.6. In Vitro Cytotoxicity of RKW on Mammalian Cells

Given the specific mechanism of action of AMPs, which involves disrupting cell membranes, they can potentially damage not only microbial cells but also normal human cells. Therefore, a key challenge in AMP research is to develop novel bioactive compounds that exhibit high selectivity toward prokaryotic cells while minimizing toxicity to host (human) cells [67].

In this context, to investigate the safety of peptide usage and assess the selective action of RKW on bacterial with respect to eukaryotic cells, its cytotoxic potential on mammalian cells was analyzed in vitro towards the mouse embryo fibroblasts BALB 3T3 clone A31 using the NRU assay, according to ISO 10993-5.

**Figure 7:**
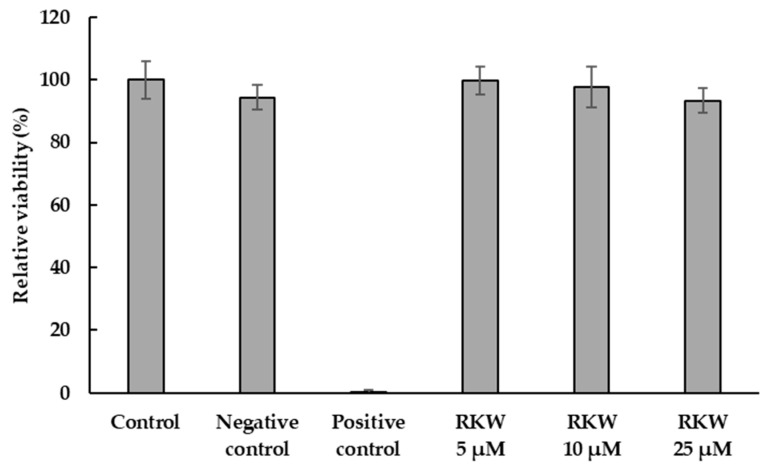
In vitro cytotoxicity of RKW on embryo fibroblasts BALB 3T3. The fibroblastic cell lines were incubated at 37 °C for 24 h in the absence (Control) or in the presence of RKW at different concentrations. Negative control: treatment medium; Positive control: SDS solution.

The addition of increasing concentrations (5, 10, and 25 µM) of RKW to cells did not result in any significant effect on viability of mammalian cells under investigation, even at the highest concentration tested, which conversely was able to kill the pathogen bacteria, indicating that the architectural differences between mammalian and bacterial membranes are mainly responsible for the selective interaction of cationic AMPs with the negatively charged prokaryotic membranes, with respect to the neutral eukaryotic ones (Figure 7). Moreover, the distribution of cationic and hydrophobic residues throughout the entire peptide and the amphipathic nature of RKW, which is closely related to the formation of an α-helical structure, affects only its antimicrobial activity in the effective concentration range without any cytotoxic effects.

To validate the results obtained from cytotoxic assay, the stability of RKW in the presence of 5% Heat-inactivated Newborn Calf Serum (Hi-NCS) was assessed, using the NCS non heat-inactivated (NCS) as a positive control. As shown in Figure S2, a clear and complete degradation pattern of RKW was observed when the NCS was not inactivated, confirming the proteolytic activity of serum proteases. Conversely, no peptide breakage was evidenced in Hi-NCS (used in NRU assay), demonstrating the stability of RKW under these experimental conditions (Figure S2).

### 3.7. In Vitro Evaluation of Bactericidal and Fungicidal Activity of RKW According to European Standard Guidelines

Despite continuing efforts, the overuse and misuse of drugs worldwide are contributing to the rapid increase in antibiotic-resistant bacteria (ARB), thus compromising the exceptional health benefits that have been reached with antibiotics [68]. However, the implementation of new policies to manage this crisis and the efforts made by the scientific world to find new agents and approaches for the treatment of bacterial infections could drastically reduce this risk. Among the numerous sources and transmission routes of ARB and infectious diseases, there are the healthcare, community, home, and industrial environments, such as the food-producing sectors. Therefore, the proper and regular environmental cleaning with wide-spectrum disinfectants represents a fundamental hygienic measure to eliminate pathogenic organisms and prevent re-infection.

Currently, microbicidal products such as chemical disinfectants and antiseptics are in routine use and lead to diminishing the number of microorganisms, including pathogenic bacteria, fungi, and viruses, thus limiting the number of infections. However, these agents have a pungent smell, strong corrosiveness, irritate the respiratory tract, damage furniture, metal objects, etc., pollute the environment to a certain extent, and most require long contact times. However, reducing the concentration of chemical disinfectants to mitigate these adverse effects significantly compromises their microbicidal effectiveness and fails to meet national disinfectant standards. Moreover, the widespread use of antiseptic/disinfectant solutions has been suggested to promote the development of antimicrobial resistance and exacerbate resistance to antibiotics [69]. Therefore, there is an urgent need to develop a kind of safe, low-toxicity, efficient, and broad-spectrum disinfectant.

In this context, the bactericidal and fungicidal activity of RKW in specific conditions of use was tested following European Standards (EN) 1276:2019 and EN 1650:2019, respectively, to evaluate the potential of the projected peptide as active ingredient in new formulations to be used in disinfection procedures, which are fundamental in various fields. In these studies, three and two reference strains of bacteria and fungi, respectively, were used. Moreover, a solution of BSA was used as an interfering substance in the test to simulate the possible contaminants that co-occur with microbes in the actual environment. The results obtained in Table 6 showed that under the tested conditions, RKW met the requirements of EUs at 20 μM concentration, where it was active against *S. epidermidis* ATCC 12228 (log R ≥ 5) and *Candida albicans* ATCC 10231(log R ≥ 4), and at 5 μM concentration against *P. aeruginosa* ATCC 15442 (log R ≥ 5).

This study could be appealing for the potential use of a new RKW-based formulation to prevent microbial contamination in several industrial sectors. Specifically, the effectiveness of RKW against fungi and both Gram-positive and Gram-negative test bacteria according to standard guidelines denotes its range of applicability against different microorganisms, enabling more specific approaches to target microbial contamination in industrial settings. Therefore, it could represent a sustainable and safe ingredient in detergent formulations, which is more environmentally friendly and effective than the commercial chemical-based products.

## 4. Conclusions

During the past half-century, the global rise in drug-resistant pathogens has made treating related diseases with existing antibiotics increasingly difficult or even impossible, complicating infection management and limiting current therapy options. In this context, AMPs have attracted considerable attention as potential next-generation antibiotics for counteracting antibiotic-resistant infections, even if associated with biofilm formation. Moreover, AMPs, beyond their direct antimicrobial action, also exhibit immunomodulatory and anti-virulence properties, influencing cytokine and chemokine production, attracting immune cells to the site of infection [70], promoting wound healing [71], neutralizing harmful bacterial toxins like lipopolysaccharides (LPS), mitigating conditions like septic shock and inflammation [70], and inhibiting virulence factors or targeting virulence mechanisms [71]. Therefore, AMPs are gaining increasing recognition for their therapeutic potential, specifically in the context of antibiotic resistance, due to their ability to disrupt multiple targets within pathogens.

In this paper, a novel cationic AMP named RKW was in silico designed by using a structure-based rational approach and evaluated for its antimicrobial efficacy. Our results indicated that the peptide was folded into an α-helical structure in membrane environments and exhibited high thermal and pH stability. Moreover, RKW demonstrated a potent antimicrobial activity against *Candida albicans*, the most prevalent pathogen causing nosocomial fungal infections, and different Gram-negative and positive bacterial strains, without cytotoxicity. Notably, the peptide also showed antibacterial effects on the most virulent and antibiotic-resistant ESKAPE pathogens and exhibited a significant antibiofilm activity against both *S. aureus* MRSA and *P. aeruginosa*.

Therefore, these findings may contribute to the ongoing efforts to develop effective strategies against the growing risk of antimicrobial resistance and provide a basis for future studies aimed at testing the effectiveness of this peptide for potential industrial and therapeutic applications, such as the development of peptide-coated medical devices to prevent microbial infections.

These investigations will also allow a more comprehensive understanding of how RKW exerts its antimicrobial effects, potentially leading to the development of new therapeutic agents. The most widely known and studied molecular mechanisms of AMPs are based on the peptide interaction with microbial cell membranes, leading to the disruption of membrane integrity, or are focused on the ability of the peptides to target intracellular molecules, which regulate essential cellular processes [70,72].

In this context, several experimental and technical approaches will be used to better investigate the RKW’s mode of action, such as (i) the dye leakage assays to assess membrane permeability; (ii) the electron or fluorescent microscopy to visualize changes in membrane morphology and integrity or tracking the localization of fluorescent-labeled peptides inside bacterial cells; and (iii) the in vitro membrane models to study physicochemical interactions between the peptide and the lipid bilayer.

## 5. Patents

Application No: 102024000012091; Publication 102024000012091A1; “Peptidi antimicrobici” BALESTRIERI Marco, PALMIERI Gianna, CAMMARNO, Aniello; MERCURIO, Maria Emilia, NICOLAIS Luigi. Status: Publication 28 May 2024.

## Figures and Tables

**Table 1:** Physical–chemical parameters of antimicrobial activity calculated for the designed peptide RKW.

Parameters	RKW RKWILKWLRTWKK
Net Charge	+6
Mol Weight	1842.31
Half-life (s)	911.91
Hydrophobicity	0.479
Hydrophobic moment	0.753
Total Hydrophobic Ratio (%)	46
Amphipathicity	1.51
GRAVY	−1.22
Wimley-White whole-residue hydrophobicity (kcal/mol)	−1.26
Hydrophilicity	0.15
Instability Index	30.26 **
Aliphatic index	90
Boman index (kcal/mol)	2.52
Total Trp ratio (%)	23.07

The physiochemical parameters of RKW were analyzed using tools from different data banks available in silico: Prot Param [37]; PlifePred [38]; Antimicrobial Peptide Database APD3 [39]; Heliquest sofware [40]. A protein whose instability index is smaller than 40 is predicted as stable (**), a value above 40 predicts that the protein may be unstable. GRAVY: Grand average hydropathy value of the peptide.

**Table 2:** RKW MBC values against pathogenic bacteria at 1.0 × 10^3^ CFU/mL concentration.

Strain	MBC (µM)
*Escherichia coli*	10
*Salmonella* Typhimurium	10
*Listeria monocytogenes*	10
*Staphylococcus aureus*	15
*Pseudomonas aeruginosa*	5
*Campylobacter jejuni*	80
Monophasic *Salmonella* Typhimurium	20
*Salmonella* Napoli	20

**Table 3:** MBC values of RKW against pathogenic bacteria at 1.0 × 10^5^ CFU/mL concentration.

Strain	MBC (µM)
*Escherichia coli*	10.0
*Salmonella* Typhimurium	10.0
*Listeria monocytogenes*	10.0
*Staphylococcus aureus*	20.0
*Pseudomonas aeruginosa*	5.0

**Table 4:** Results of antimicrobial susceptibility per four ESKAPE bacteria testing by the disk diffusion method.

Bacteria	Antibiotic Class	Antibiotic	Disc Content (μg)	R * < (mm)	Inhibition Zone Diameter (mm)	Results
*Acinetobacter baumannii*	Aminoglycosides	Amikacin	30	19	-	R
		Gentamicin	10	17	-	R
		Tobramycin	10	17	-	R
	Carbapenems	Imipenem	10	21	16	R
		Meropenem	10	21	10	R
	Fluoroquinolones	Ciprofloxacin	5	21	-	R
		Levofloxacin	5	20	-	R
	Miscellaneous agents	Trimethoprim-sulfamethoxazole	1.25–23.75	11	-	R
*Enterococcus faecium*	Carbapenems	Imipenem	10	21	50	S
	Fluoroquinolones	Ciprofloxacin	5	15	-	R
		Levofloxacin	5	15	-	R
	Glycopeptide	Vancomycin	5	12	-	R
	Penicillins	Ampicillin	2	10	-	R
*Staphylococcus aureus* (MRSA)	Aminoglycosides	Amikacin	30	15	25	S
		Gentamicin	10	18	28	S
		Tobramycin	10	18	29	S
	Cephalosporins	Cefoxitin	30	17	15	R
	Fluoroquinolones	Ciprofloxacin	5	17	-	R
		Levofloxacin	5	22	-	R
	Macrolides	Erythromycin	15	21	-	R
	Tetracyclines	Tetracycline	30	22	32	S
	Miscellaneous agents	Trimethoprim-sulfamethoxazole	1.25–23.75	14	30	S

R: resistant; S: susceptible; *: EUCAST breakpoints.

**Table 5:** Results of antimicrobial activity of RKW against four ESKAPE bacteria.

Bacterial Inoculum Concentration	5 × 10^3^ CFU/mL		5 × 10^5^ CFU/mL	
	MIC	MBC	MIC	MBC
*Acinetobacter baumannii* (CRAB)	50 μM	50 μM	75 μM	>75 μM
*Enterococcus faecium* (VR*Efm*)	20 μM	20 μM	20 μM	50 μM
*Pseudomonas aeruginosa*	20 μM	20 μM	75 μM	>75 μM
*Staphylococcus aureus* (MRSA)	50 μM	50 μM	50 μM	50 μM

**Table 6:** Test procedure of the evaluation of bactericidal or fungicidal activity of chemical disinfectants and antiseptics used in food, industrial, domestic, and institutional areas.

Bacteria	Test Suspension	Peptide Concentration (µM)				
		100	50	20	10	5
*Staphylococcus* *aureus * ATCC 6538	N: 2.00 × 10^8^ N_0_: 2.00 × 10^7^ log N_0_: 7.30	N_a_ = 1.47 × 10^4^ log N_a_ = 4.20 log R = 3.13	N_a_ = 1.47 × 10^4^ log N_a_ = 4.20 log R = 3.13	N_a_= 1.47 × 10^4^ log N_a_ = 4.20 log R = 3.13 **Not active**	N_a_ = 5.10 × 10^4^ log N_a_ = 4.71 log R = 2.59 **Not active**	N_a_ = 1.11 × 10^5^ log N_a_ = 5.10 log R = 2.25 **Not active**
*Pseudomonas * *aeruginosa * ATCC 15442	N: 2.50 × 10^8^ N_0_: 2.50 × 10^7^ log N_0_: 7.40	N_a_ < 1.40 × 10^2^ log N_a_ < 2.15 log R > 5.25 **Active**	N_a_ < 1.40 × 10^2^ log N_a_ < 2.15 log R > 5.25 **Active**	N_a_ < 1.40 × 10^2^ log N_a_ < 2.15 log R > 5.25 **Active**	N_a_ < 1.40 × 10^2^ log N_a_ < 2.15 log R > 5.25 **Active**	N_a_ < 1.40 × 10^2^ log N_a_ < 2.15 log R > 5.25 **Active**
*Staphylococcus * *epidermidis * ATCC 12228	N: 1.90 × 10^8^ N_0_: 1.90 × 10^7^ log N_0_: 7.28	N_a_ = 1.60 × 10^2^ log N_a_ = 2.20 log R = 5.08 **Active**	N_a_ = 1.60 × 10^2^ log N_a_ = 2.20 log R = 5.08 **Active**	N_a_ = 1.60 × 10^2^ log N_a_ = 2.20 log R = 5.08 **Active**	N_a_ = 2.40 × 10^2^ log N_a_ = 2.38 log R = 4.90 **Not active**	N_a_ = 3.70 × 10^2^ log N_a_ = 2.57 log R = 4.71 **Not active**
**Fungi**						
*Candida * *albicans * ATCC 10231	N: 3.95 × 10^7^ N_0_: 3.95 × 10^6^ log N_0_: 6.60	N_a_ = 2.75 × 10^2^ log N_a_ = 2.44 log R = 4.16 **Active**	N_a_ = 2.75 × 10^2^ log N_a_ = 2.44 log R = 4.16 **Active**	N_a_ = 2.75 × 10^2^ log N_a_ = 2.44 log R = 4.16 **Active**	N_a_ = 1.08 × 10^3^ log N_a_ = 3.03 log R = 3.57 **Not Active**	N_a_ = 7.70 × 10^4^ log N_a_ = 4.89 log R = 1.71 **Not Active**
*Aspergillus * *brasiliensis * ATCC 16404	N: 3.95 × 10^7^ N_0_: 3.95 × 10^6^ log N_0_: 6.60	N_a_ = 2.20 × 10^6^ log N_a_ = 6.34 log R = −0.09 **Not Active**	N_a_ = 2.20 × 10^6^ log N_a_ = 6.34 log R = −0.09 **Not Active**	N_a_ = 2.20 × 10^6^ log N_a_ = 6.34 log R = −0.09 **Not Active**	N_a_ = 3.30 × 10^6^ log N_a_ = 6.52 log R = −0.29 **Not Active**	N_a_ = 3.40 × 10^6^ log N_a_ = 6.53 log R = −0.28 **Not Active**

N = number of CFU/mL in the test suspension. N_0_= number of CFU/mL in the test mixture at the beginning of the contact time. N_a_ = number of CFU/mL in the test mixture at the end of the contact time. R = reduction in viability (log R = log N_0_ − log Na). For bacteria: ACTIVE if log R ≥ 5 according to BS EN 1276:2019. For fungi: ACTIVE if log R ≥ 4 according to BS EN 1650:2019.

## Data Availability

The raw data supporting the conclusions of this article will be made available by the authors on request.

## Supplementary Materials

The following supporting information can be downloaded at: https://www.mdpi.com/article/10.3390/biom15070989/s1, Figure S1: Bactericidal activity of RKW against *S. aureus*, *L. monocytogenes*, *P. aeruginosa*, *E. coli* and *S*. Typhimurium. Figure S2: Stability of RKW in Newborn Calf Serum (NCS) determined by RP-HPLC chromatography.
