# Supplementary material for: Discovery of a Potent Antimicrobial Peptide Through Rational Design: A New Frontier in Pathogen Control
Source: Biomolecules. 2025 Jul 11;15(7):989. doi: 10.3390/biom15070989 (PMC12292521; doi:10.3390/biom15070989)
Supplement: Supplementary file 1 [file biomolecules-15-00989-s001.zip › biomolecules-3685215-supplementary.pdf]

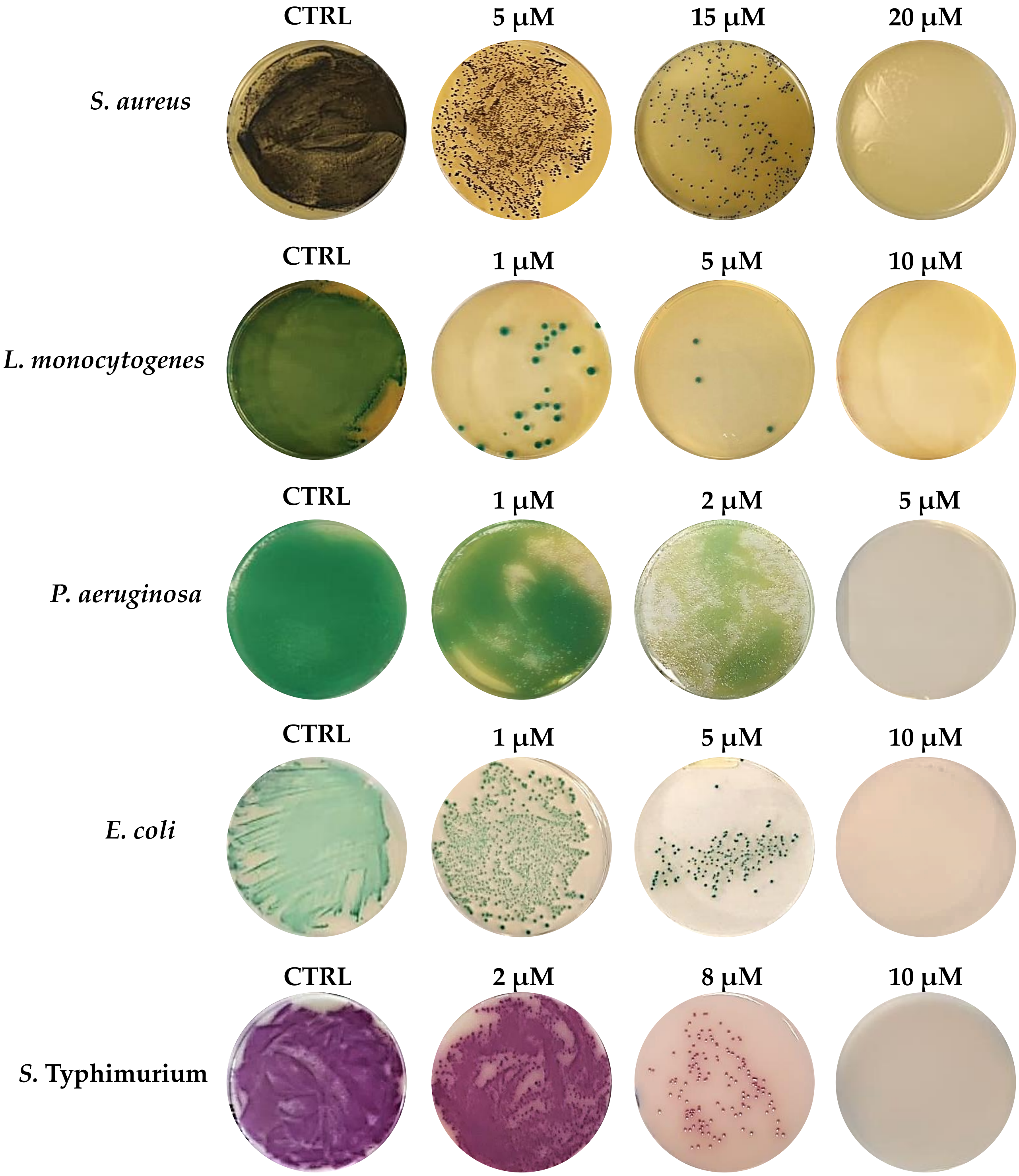

Figure S1. Bactericidal activity of RKW against *S. aureus*, *L. monocytogenes*, *P. aeruginosa*, *E. coli* and *S.* Typhimurium. Bacterial cultures untreated (CTRL) or treated with peptide at different concentrations for 6 h were seeded on selective plates. The photographs are representative of three independent experiments performed in triplicate Data were determined by counting the surviving colony-forming units (CFU) on plates seeded with the pathogens. The bacterial suspensions were diluted in fresh broth to a final concentration of 1.0 × 10^5 CFUs/mL

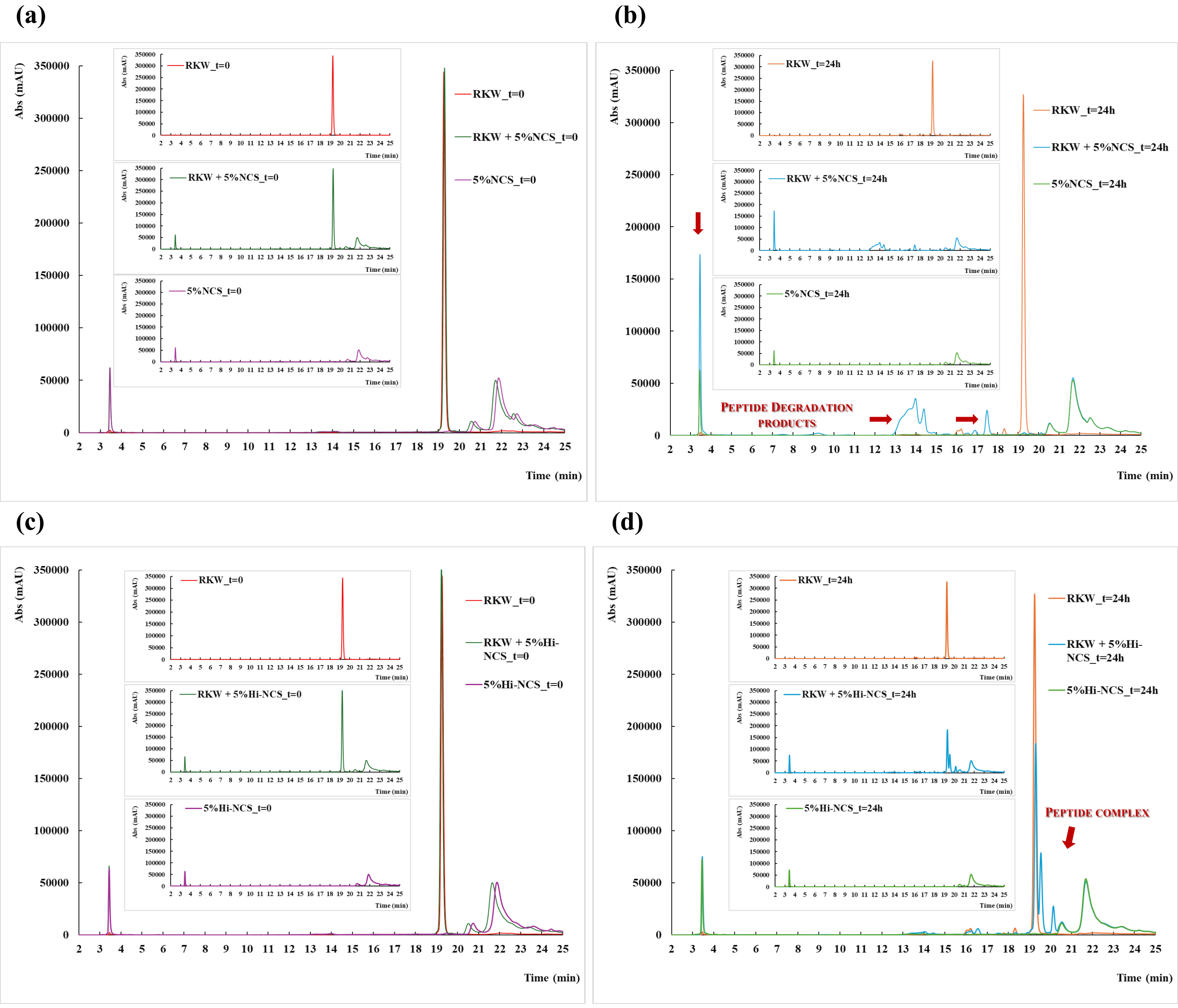

Figure S2. Stability of RKW in Newborn Calf Serum (NCS) determined by RP-HPLC chromatography. RKW at 100 μM was incubated for 24 h at 37 °C in the presence of 5% NCS, heat-inactivated (Hi-NCS) or not (NCS) at 57 °C for 45 min. After incubation, the reactions were stopped with 95% Acetonitrile/0.1%TFA. Therefore, the samples were centrifuged, the supernatants collected, and 200 μL analysed by RP-HPLC on a C18 column. **(a)** RKW, RKW in the presence of 5% NCS, and 5% NCS at t=0; **(b)** RKW, RKW in the presence of 5% NCS, and 5% NCS after 24 h incubation (t=24 h); **(c)** RKW, RKW in the presence of 5% Hi-NCS, and 5% Hi-NCS at t=0; **(d)** RKW, RKW in the presence of 5% Hi-NCS, and 5% Hi-NCS after 24 h incubation (t=24 h). The reported chromatograms are representative of three independent experiments.
